# Role of macrophage in intervertebral disc degeneration

**DOI:** 10.1038/s41413-024-00397-7

**Published:** 2025-01-23

**Authors:** Yiming Dou, Yiming Zhang, Yang Liu, Xun Sun, Xinyu Liu, Bin Li, Qiang Yang

**Affiliations:** 1 Department of Spine Surgery, Tianjin Hospital, Tianjin University, Tianjin, 300211 China; 2 Clinical School of Orthopedics, Tianjin Medical University, Tianjin, 300070 China; 3 Department of Orthopaedics, Qilu Hospital of Shandong University, Jinan, 250012 China; 4 Orthopedic Institute, Department of Orthopedic Surgery, The First Affiliated Hospital, School of Biology & Basic Medical Sciences, Suzhou Medical College, Soochow University, Suzhou, Jiangsu 215007 China

**Keywords:** Homeostasis, Bone, Bone quality and biomechanics

## Abstract

Intervertebral disc degeneration is a degenerative disease where inflammation and immune responses play significant roles. Macrophages, as key immune cells, critically regulate inflammation through polarization into different phenotypes. In recent years, the role of macrophages in inflammation-related degenerative diseases, such as intervertebral disc degeneration, has been increasingly recognized. Macrophages construct the inflammatory microenvironment of the intervertebral disc and are involved in regulating intervertebral disc cell activities, extracellular matrix metabolism, intervertebral disc vascularization, and innervation, profoundly influencing the progression of disc degeneration. To gain a deeper understanding of the inflammatory microenvironment of intervertebral disc degeneration, this review will summarize the role of macrophages in the pathological process of intervertebral disc degeneration, analyze the regulatory mechanisms involving macrophages, and review therapeutic strategies targeting macrophage modulation for the treatment of intervertebral disc degeneration. These insights will be valuable for the treatment and research directions of intervertebral disc degeneration.

## Introduction

**Fig. 1:**
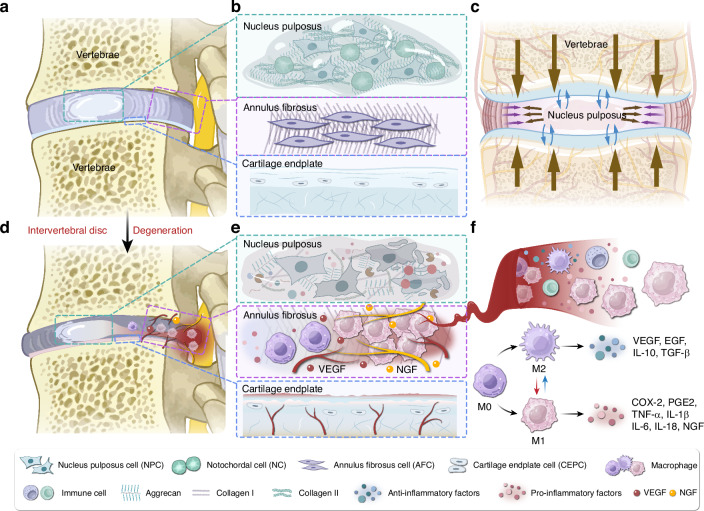
Schematic diagram of healthy IVD, degenerated IVD, and inflammatory pathological microenvironment. **a** Overview of the IVD. The IVD consists of three parts: the NP, the AF, and the CEP. **b** Cells and ECM in healthy IVD. In healthy IVD, the NP mainly consists of notochordal cells and nucleus pulposus cells. The AF primarily consists of annulus fibrosus cells. The CEP mainly consists of chondrocytes. **c** Mechanical function of the IVD. The primary function of the IVD is to provide mechanical support for the vertebrae and allow movement of the spine (flexion, extension, and rotation). **d** IVDD and IVD herniation. Severe IVDD leads to NP degeneration, AF rupture, and endplate calcification, resulting in IVD herniation that compresses the spinal cord and nerve roots. **e** Change of cells and ECM in degenerated IVD. In degenerated IVD, notochordal cells disappear, and due to cell senescence and PCD, the number of IVD cells significantly decreases, and their function markedly declines. **f** Macrophage infiltration and polarization. Under the induction of chemokines, macrophages infiltrate the IVD via neovascularization. Macrophages can polarize into M1 and M2 subtypes under different stimuli

Low back pain (LBP) has emerged as the leading cause of disability, resulting in substantial labor and economic losses.^1^ Intervertebral disc degeneration (IVDD) is one of the main causes of LBP.^2^ Intervertebral disc (IVD) is a fibrous cartilaginous tissue connecting two adjacent vertebral bodies and consists of three parts: nucleus pulposus (NP), annulus fibrosus (AF), and cartilage endplate (CEP) (Fig. 1a). NP is a centrally located, highly hydrated gel-like tissue, encased by the AF. CEP is a hyaline cartilage structure that connects the vertebral bodies to the IVD. The blood supply ends at CEP, making it responsible for substance exchange and nutrient transport within the IVD^3,4^ (Fig. 1b). These three distinctly different tissues form a sealed microenvironment within the IVD, rendering it both avascular and immune-privileged.^5^ IVD plays a crucial role in the transmission and absorption of mechanical loads on the spine, thereby maintaining motor function (Fig. 1c).

IVDD is a complex degenerative disease involving multiple pathological processes. Frapin et al. outlined three pathological events in IVDD: (1) inflammation and catabolic cascades, (2) continuous loss of cells, and (3) decline in cellular functions and anabolic activities.^6^ Inflammation and cytokine imbalance are central to IVDD, as the balance between pro-inflammatory and anti-inflammatory factors is disrupted, aggravating the degenerative process. Cellular loss encompasses the disappearance of notochordal cells, which are vital for the disc’s microenvironment, and a reduction in the number of disc cells due to abnormal programmed cell death (PCD). Cellular senescence leads to a decline in the function of IVD cells. Extracellular matrix (ECM) metabolic imbalance is characterized by excessive degradation and reduced synthesis of ECM components. These processes are interlinked and together result in the structural and functional deterioration of the IVD.

Degeneration of NP is often an early manifestation of IVDD. Current research suggests that IVDD begins with the loss of notochordal cells in the NP, leading to its dysfunction.^4^ This dysfunction causes biomechanical imbalances in IVD, affecting the surrounding AF and CEP, further exacerbating overall degeneration. Concurrently, the progression of IVDD is accompanied by damage to the AF and CEP, resulting in neovascularization and nerve growth in the damaged areas, which leads to macrophage-dominated immune cell infiltration^7^ (Fig. 1d). More importantly, the physical barrier between IVD and immune system becomes damaged, which exposing the NP to the immune system. Then, the systemic immune system will recognize the “immune-privileged” NP as a “foreign antigen” and induce the initial immune response (primary response).^8–10^ The products of these immune cells, such as interleukins (ILs), tumor necrosis factor-alpha (TNF-α), interferon-gamma (IFN-γ), and matrix metalloproteinases (MMPs), lead to a reduction in cell numbers and a deterioration of the IVD microenvironment.^11^ Recent studies indicate that elevated macrophage accumulation within the IVD correlates with increasing levels of disease severity.^12^ N. Djuric et al. identified mixed phenotype macrophage populations in human herniated discs, with the infiltration of these macrophages closely associated with the extruded type of disc herniation, potentially leading to suboptimal inflammatory peripheral neuropathy, as seen in sciatica.^13^

Macrophages begin to participate in the progression of IVDD early in the degeneration process, being involved in almost all pathological events of IVDD and having a profound impact on the exacerbation of IVDD. Macrophages can influence the progression of IVDD in two ways. First, following damage to the AF, macrophages can easily enter the IVD through neovascularization, rapidly amplifying the inflammatory response and dominating the inflammatory microenvironment, thus participating in nearly all pathological events in IVDD^14,15^ (Fig. 1e). On the other hand, macrophages from various sources, including peripheral blood and adjacent tissues, contribute to the inflammatory response and repair processes following endplate damage and degeneration.^16–18^ This continuous cycle of damage and repair at the endplate exacerbates its degeneration, resulting in impaired material exchange and mechanical balance between the endplate and the NP, which can trigger Modic changes and further endplate lesions. Macrophages also affect a series of cellular activities in disc cells, including proliferation, senescence, and PCDs, ultimately leading to IVD dysfunction by interfering with the ECM metabolism of IVD cells.

IVDD is inevitable, and currently, clinical treatments for IVDD primarily consist of pharmacological and surgical interventions. While these methods can improve symptoms to some extent, they cannot reverse disc degeneration.^4^ Therefore, finding ways to treat IVDD by regulating its pathological microenvironment is of great clinical significance. Given the significant link between macrophages and IVDD, there has been growing interest over the past few years in the impact of macrophages on IVDD progression, and research into treating IVDD by mediating macrophage polarization has shown a dramatic trend. What role do macrophages play in the development of IVDD? How can interventions targeting macrophages regulate the inflammatory microenvironment of IVDD? In this review, we will analyze the role of macrophages within the pathological events of IVDD and summarize recent therapeutic strategies targeting macrophages, offering insights into treating IVDD from the perspective of inflammation regulation.

## Involvement of macrophages in pathological events of IVDD

Macrophages, essential immune cells derived from monocytes in the bone marrow, are involved in both specific and nonspecific immunity, playing a vital role in the inflammatory response, defense strategies, repair functions, and metabolic processes.^19^ Macrophage activation and polarization are influenced by various initial factors, primarily pro-inflammatory cytokines and microbial products.

The polarization of M1 macrophages in IVD may be closely related to the excessive activation of the NF-κB pathway in inflammatory IVD cells. Burt et al. observed increased F4/80^+^/CD38^+^ (M1) and F4/80^+^/CD206^+^ (M2) macrophages in the caudal IVDs of NF-κB-overactivated mice (IKKβCA mice). Secretome analysis revealed that conditioned media (CM) from IKKβCA IVDs contained significantly higher levels of IL-1β, IL-6, IFN-γ, and MCP-1 compared to controls. Further experiments showed that this altered secretome promoted macrophage migration and M1 polarization while suppressing M2 polarization.^20^ Chen et al. conducted single-cell sequencing analysis of human NP tissue and identified a subpopulation of late-stage degenerative nucleus pulposus cells (NPCs) with high serglycin expression. Further studies revealed that serglycin secreted by these cells promotes inflammatory cytokine secretion via activation of the NF-κB pathway. Pathway and ligand-receptor analyses, combined with cellular experiments, demonstrated that serglycin-high cells exacerbate local inflammation by promoting macrophage infiltration and M1 polarization.^21^ Wang et al. reported that osteopontin (OPN), which is primarily expressed in the CEP, decreases during degeneration in both mice and human patients with severe IVDD. OPN deficiency leads to increased production of CCL2 and CCL5 in cartilage endplate cells (CEPCs), promoting macrophage recruitment and enhancing NLRP3 inflammasome activation and NF-κB signaling via assembly of the IRAK1-TRAF6 complex, thereby exacerbating CEP degeneration.^17^ Exosomes also serve as key mediators of communication between macrophages and IVD cells. Zhao et al. found that exosomes derived from degenerative NPCs significantly induce M1 polarization of macrophages compared to those from healthy NPCs. RNA sequencing revealed that miR-27a-3p is highly expressed in exosomes from degenerative NPCs. Further research confirmed that these exosomes induce M1 polarization of macrophages by transferring miR-27a-3p, which targets the PPARγ/NF-κB/PI3K/AKT signaling pathway.^22^

On the other hand, M2 macrophage polarization may be closely associated with TGF-β expression in the IVD. Yokozeki et al. reported that compared to young mice, aged mice exhibited significantly reduced expression of CD206 (M2 macrophage marker) and TGF-β in their IVDs. Further studies revealed that TGF-β stimulation significantly increased CD206 expression in disc macrophages from both young and aged mice. Conversely, administration of a TGF-β inhibitor significantly reduced CD206 expression in both groups, suggesting that M2 macrophage polarization in IVDs is driven by TGF-β activation.^23^ Kawakubo et al. similarly demonstrated that injecting mice with the TGF-β inhibitor SB431542 reduced both CD206-positive cells and CD 206 expression.^24^ Additionally, Gao et al. discovered that human NPCs express CX3C chemokine ligand 1 (CX3CL1), which binds to the CX3C motif chemokine receptor 1 (CX3CR1) on macrophages, promoting M2 phenotype polarization via the JAK2/STAT3 signaling pathway.^25^

Feedback control mechanisms between key signaling pathways help maintain the balance between M1 and M2 macrophage phenotypes, influencing the progression and resolution of inflammatory diseases.^26^ M1 macrophages are known for their pro-inflammatory cytokine secretion, participating in host defense by producing reactive oxygen species (ROS), TNF-α, IL-1, IL-6, IL-18, and various chemokines. They primarily mount an inflammatory response against microbial invasion, support host immunity, and can potentially harm healthy tissue. In contrast, M2 macrophages secrete TGF-β, IL-10, vascular endothelial growth factor (VEGF), epidermal growth factor (EGF), and similar agents, marking a transition to an anti-inflammatory function that becomes particularly evident in the later stages of inflammation. M2 macrophages are also significantly involved in wound healing and the fibrotic process.^27^

### Macrophages infiltration and heterogeneity in IVD

Macrophage infiltration refers to the process where macrophages accumulate and aggregate extensively within tissues or organs. When tissues are stimulated by infection, injury, or inflammation, macrophages are attracted to the affected areas. They are guided to the lesion site by chemotactic factors and other signaling molecules, where they engulf and degrade pathogens or damaged cells, aiding in the clearance of inflammatory sources and the restoration of tissue function. Macrophages also secrete various cytokines to regulate inflammatory and immune responses, helping to maintain the balance of the immune system.^28,29^ However, for the IVD, an immune-privileged organ, excessive macrophage infiltration can lead to over-inflammation or adverse outcomes. Persistent inflammatory stimuli are a critical factor in the progression of IVDD. CD68^+^ cells are often detected in degenerated IVD, indicating macrophage infiltration.^30^

Research has shown that degenerated IVD cells are capable of producing a variety of chemokines, such as monocyte chemoattractant proteins (MCP-1, MCP-3, MCP-4), chemokine (CC motif) ligands (CCL3, CCL4, CCL5), interferon-γ–inducible protein-10 (IP-10), colony stimulating factor 2 (CSF2), and CXC chemokine ligand 8 (CXCL8). When the AF fissures, these chemokines attract and activate immune cells, including macrophages, T cells, and B cells to the degenerated sites. This infiltration leads to further secretion of cytokines in an autocrine manner, enhancing immune cell recruitment and activation, thereby exacerbating the degeneration of the IVD.^11,31–33^ During the inflammatory process, both M1 and M2 macrophages are intimately involved and play key roles in regulating cellular activities of IVD cells, maintaining inflammatory balance, tissue repair, tissue remodeling, pain, and angiogenesis.^12^

As previously noted, once exposed, NP is recognized by the immune system as an antigen, triggering an immune response. During immune cell infiltration, macrophages from various tissue sources are involved in IVDD to varying extents. Specifically, macrophages in different regions of IVD and at different stages of IVDD exhibit dynamic changes in polarity, distribution, and origin, indicating macrophage heterogeneity.

Firstly, there is heterogeneity in polarity: different subtypes of macrophages dominate IVDD inflammation at different degeneration stages. Nakawaki et al. reported a significant increase in macrophages on day 1 post-IVD injury in mice, with M1 macrophages predominating initially. Subsequently, the proportion of M1 macrophages gradually decreased, while the proportion of M2 macrophages significantly increased on days 7, 14, and 28 post-disc injury.^34^ Jin et al. also found abundant heterogeneous macrophage populations at disc hernia sites 7 days post-disc puncture modeling,^35^ suggesting a polarization change following macrophage infiltration. This aligns with observations from human IVD samples. Nakazawa et al. observed a significant increase in M1 macrophages in damaged, unhealthy disc areas.^36^ Ling et al. noted an increased number of inflammatory response NPCs along with M1 macrophages in grade IV IVD compared to grade II, indicating that M1 macrophages play a primary role in inflammation.^37^ With further IVDD progression, M2 macrophages may shift to become the dominant subgroup.^15^ Djuric et al. collected IVD samples from patients undergoing surgery for cervical or lumbosacral radiculopathy. After immunohistochemical processing and macrophage marker staining, they found that the expression of M2 markers CD163 and CD209 was most prominent, followed by the M1 marker CD192, suggesting that anti-inflammatory macrophages are predominant in symptomatic lumbar and cervical disc herniations rather than pro-inflammatory macrophages.^38^

Secondly, there is heterogeneity in the distribution of different macrophage subtypes in various disc regions. Li et al. explored the distribution of macrophage subtypes in different regions of IVD samples from patients with lumbar disc herniation and found that the proportions of M1 macrophages were higher in high-intensity zones (HIZ), as well as in bulging and herniated NP tissues, compared to non-HIZ and protruding NP tissues. A higher proportion of M2 macrophages was detected in cases with Modic changes, suggesting that pro-inflammatory phenotypes dominate in IVDD primarily involving annulus fibrosus injury, while anti-inflammatory phenotypes increase with endplate degeneration.^39^

Lastly, there is heterogeneity in macrophage origins across various IVD regions. Studies suggest that disc macrophages may derive from bone marrow-derived macrophages recruited through peripheral blood or from resident macrophages in nearby adipose or muscle tissue. Kawakubo et al. used GFP chimeric mice and induced IVD injury by puncture. Flow cytometry results showed a significant increase in CD86^+^ macrophages in the GFP^+^ group on days 1, 3, 7, and 14 post-puncture. On days 7 and 14 post-puncture, CD206-positive cells increased significantly in the GFP- group, indicating that CD86^+^ pro-inflammatory M1 macrophages rapidly increased after disc injury, while CD206-positive anti-inflammatory M2 macrophages gradually increased from day 7 onward. This suggests that infiltrated M1 and M2 macrophages in IVDD may originate from different sources: bone marrow-derived macrophages and resident macrophages, respectively.^24^

Different subtypes of macrophages can communicate with IVD cells through cytokines, chemokines, and extracellular vesicles, influencing each other’s fate by triggering various molecular mechanisms (Table 1). The ratio of M1 to M2 macrophages in clinical specimens also provides insights into the disease state or progression in IVDD. Therefore, regulating the phenotypic transition of infiltrated macrophages (M1 pro-inflammatory and M2 anti-inflammatory subtypes) is crucial for controlling tissue inflammation.

**Table 1:** Mechanisms of cell communications between macrophages and IVD cells

Source	Component	Target cell	Mechanism	Outcomes	References
M1	IL-1β	NPC	Stimulate NF-κB signaling pathway	Promote the expression of inflammatory cytokines and chemokines	^40,56^
		NPC	Stimulate MAPK signaling pathway	Promote the expression of inflammatory cytokines	^133^
		NPC	Suppress PI3K/Akt signaling pathway	Inhibit cell proliferation, promote apoptosis and senescence	^92^
		NPC	Stimulate JAK/STAT3 signaling pathway	Promote apoptosis and ECM degradation	^253^
		AFC	Stimulate ERK pathway	Induces apoptosis	^254^
		AFC	Stimulate NF-κB signaling pathway	Promote inflammation and ECM degradation	^93^
		CEPC	Stimulate PI3K/Akt signaling pathway	Inhibit cell proliferation and induce apoptosis, inhibited the synthesis and metabolism of CEPC	^255^
		CEPC	Stimulate Wnt/β‐catenin signaling pathway	Inhibit the expression of Collagen II, ACAN and Sox-9. Promote the expression of ADTAMTS5 and MMP13	^256^
	TNF-α	NPCs	Stimulate MAPK signaling pathway	Promote the expression of CHOP and promote apoptosis	^99^
		NPCs	Stimulate NF-κB signaling pathway	Promote the expression of inflammatory cytokines and chemokines, Promote pyroptosis and cell senescence	^56,98,257^
		NPCs	Stimulate JAK/STAT3 signaling pathway	Promote cell senescence	^115^
		NPCs	Stimulate UPR signaling pathway	TNF-α stimulus upregulated ER stress markers and initiated unfolded protein response (UPR), promoting cell proliferation	^126^
		AFC	Stimulate NF-κB signaling pathway	Inhibit cell proliferation, promote cell senescence and the expression of ROS	^258^
	IL-18	NPC	Stimulate Caspase-3/9 signaling pathway	Promote apoptosis and ECM degradation	^60^
	IL-6	NPC	Stimulate JAK/STAT3 signaling pathway	Promote the expression of COX-2, MMP-13 and catabolic activity	^45,259^
		NPC	Stimulate YAP1/β-catenin signaling pathway	Inhibit the expression of Sox-9, Col-II and ACAN, and promote the expression of MMP 13	^138^
	CXCL8	NPC	Stimulate NF-κB pathway signaling pathway	Promote apoptosis, oxidative stress, inflammation and ECM degradation	^260^
	Exosome	NPC	Stimulate LCN2/NF-κB signaling axis	Promote cell senescence	^261^
	ROS	NPC	Stimulate MAPK signaling pathway	Promote cell senescence	^262^
		NPC	Stimulate NF- κ B signaling pathway	Promote cell senescence	^262^
		NPC	Stimulate Hippo-p53 signaling pathway	Promote cell senescence	^263^
M2	IL-10	NPC	Suppress MAPK signaling pathway	Induce anti-inflammatory response and delay IVDD	^75^
		EC	Stimulate pSTAT3 signaling	Promote endplate vascularization and sclerosis	^18^
	TGF-β	NPC	Suppress MAPK signaling pathway	Inhibit catabolic activity and inflammation	^264^
		NPC	Stimulate TGF-β/Smad signaling pathway	Inhibit ECM degradation and promote ECM synthesis	^78^
		NPC	Suppress NF-κB signaling pathway	Promote cell proliferation, inhibit inflammation and oxidative stress	^86^
	Exosome	NPC	Stimulate TGF-β/smad3 signaling pathway	Promote the expression of Col-II and ACAN, inhibit the expression of MMP13 and ADAMTs5 by delivering miR-124-3p	^139^
	Small EV	NPC	Suppress PTEN/NLRP3 signaling pathway	Inhibit pyroptosis by delivering miR-221-3p	^265^
NPC	CCL2/7	Macrophage	CCL2/7-CCR2 axis	Promote macrophage infiltration	^266–268^
	CX3CL1	Macrophage	CX3CL1-CX3CR1	Promote M2 polarization	^25^
	CCL3	Macrophage	CCL3-CCR1	Promote macrophage infiltration	^51^
	CCL4	Macrophage	CCL4-CCR1	Promote macrophage infiltration	^269^
	Exosome	Macrophage	Stimulate PPARγ/NFκB/PI3K/AKT signaling pathway	Promote M1 polarization	^22^

*M1* M1 macrophage, *M2* M2 macrophage, *NPC* Nucleus pulposus cell, *AFC* Annulus fibrosus cell, *CEPC* Cartilage endplate cell, *MMP* matrix metalloproteinase, *TNF-α* tumor necrosis factor-alpha, *IL* interleukin, *MAPK* Mitogen-activated protein kinase, *NF-κB* Nuclear factor kappa-light-chain-enhancer of activated B cells, *JAK* Janus kinase, *STAT* Signal transducer and activator of transcription, *PI3K* Phosphoinositide 3-kinase, *Akt* Protein kinase B, *ERK* Extracellular signal-regulated kinase, *COX* Cyclooxygenase, *YAP* Yes-associated protein, *TGF-β* Transforming growth factor-beta, *Smad* Sma and mad-related protein, *PTEN* Phosphatase and tensin homolog, *NLRP3* NOD-like receptor pyrin domain containing 3, *PPARγ* Peroxisome proliferator-activated receptor gamma, *ROS* reactive oxygen species, *IVDD* intervertebral disc degeneration, *LCN2* Lipocalin 2, *pSTAT3* Phosphorylated signal transducer and activator of transcription 3, *CCL* C-C motif chemokine ligand, *CCR* C-C chemokine receptor, *CX3CR1* C-X3-C motif chemokine receptor 1, *Small EV* small extracellular vesicle, *UPR* unfolded protein response, *ECM* extracellular matrix

### Macrophages regulate the inflammatory balance in IVDD

**Fig. 2:**
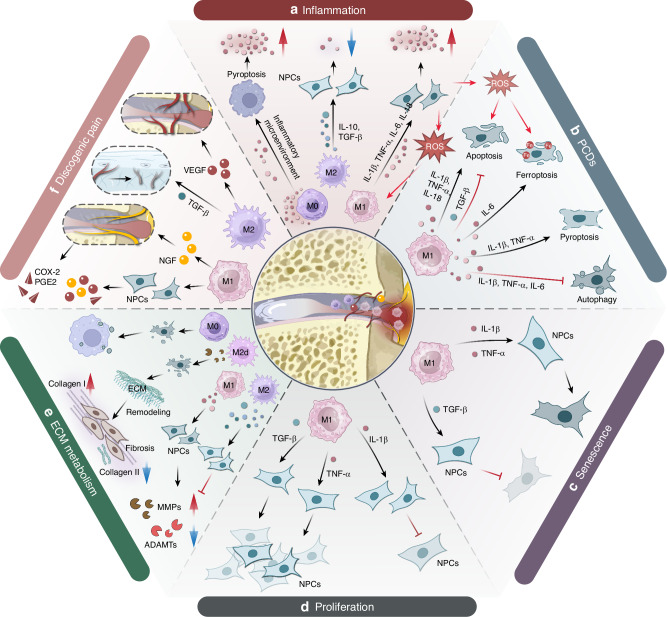
Role of macrophages in IVDD. **a** Macrophages amplify inflammation in the IVD. Macrophages upregulate the expression of pro-inflammatory cytokines, creating an inflammatory microenvironment and causing a series of effects on cellular activities and ECM metabolism. **b** Macrophage-related cytokines regulate PCDs of IVD cells, causing a significant loss of IVD cells. **c** Macrophages accelerate the senescence of IVD cells. **d** Macrophages regulate the proliferation of IVD cells. **e** Macrophages regulate ECM metabolism. Macrophages phagocytize disc fragments and regulate ECM metabolism by upregulating the expression of MMPs and ADAMTs. This activity can lead to the resorption of herinated NP or exacerbate the progression of IVDD. **f** Macrophages regulate discogenic LBP. Macrophages can promote nerve growth by upregulating NGF, increasing IVD sensitization, and causing discogenic LBP through inflammatory factors. Macrophages can also promote the formation of new blood vessels in the IVD by upregulating VEGF expression, bringing more immune cells and exacerbating the immune response

Inflammation plays a central role in IVDD, and after macrophage infiltration, the inflammatory response in IVDD is rapidly amplified. During the inflammatory process, macrophages primarily regulate the inflammatory environment through the secretion of cytokines, thereby affecting immune responses and tissue repair. As the main pro-inflammatory regulators, M1 macrophages secrete a variety of cytokines that promote the progression of inflammation. Key pro-inflammatory cytokines that play a major role in IVDD include TNF-α, IL-1, IL-6, and IL-18.^40–42^ These factors are involved in the activation of pathways such as nuclear factor kappa-light-chain-enhancer of activated B cells (NF-κB),^43^ mitogen-activated protein kinases (MAPKs),^44^ Janus kinase-signal transducer and activator of transcription (JAK-STAT),^45^ toll-like receptor (TLR),^46^ as well as the assembly and activation of the NLRP3 inflammasome,^47–49^ triggering inflammatory responses. These pro-inflammatory factors stimulate IVD cells to secrete more pro-inflammatory factors and chemokines, forming a positive feedback loop that recruits more macrophages. This results in an inflammatory cascade, continuously enhancing the local inflammatory microenvironment and leading to chronic inflammation and more severe IVDD (Fig. 2a).

TNF-α is one of the most critical pro-inflammatory cytokines in the progression of IVDD. James et al. conducted immunofluorescence assays on multifidus muscle tissue in a sheep IVD injury model. They found that a greater proportion of M1 macrophages was present in muscle at both 3 and 6 months post-IVD lesion, and in adipose tissue at 6 months. By 6 months, the expression of TNF-α is increased in adipose and connective tissues, and the proportion of TNF-α expressed by M1 macrophages was also elevated. This suggests that M1 macrophages may promote inflammatory responses in the IVD through the secretion of the pro-inflammatory cytokine TNF-α.^50^ TNF-α stimulates IVD cells to produce more inflammatory cytokines, including IL-1, IL-6, IL-8, IL-17, nitric oxide (NO), prostaglandin E2 (PGE2), and chemokines, further exacerbating the inflammatory environment within the IVD.^51–53^

IL-1β is a critical cytokine that regulates the innate immune response.^41,54^ Chen et al. reported that the expression of IL-1β was higher in degenerative IVD and found a positive correlation with degeneration scores.^55^ Like TNF-α, IL-1β can induce the production of IL-6, IL-8, TNF-α, PGE2, and IL-17.^56^ Wang et al. demonstrated that TNF-α or IL-1β could promote macrophage migration by stimulating NPCs to express CCL3.^51^ Yoshida et al. observed that both NPCs and annulus fibrosus cells (AFCs) produced MCP-1 in a dose- and time-dependent manner following stimulation by IL-1β and TNF-α, thereby recruiting more macrophages.^57^ The recruited macrophages further contribute to IVDD. Additionally, the inflammatory environment induces macrophage apoptosis and pyroptosis, resulting in the release of large amounts of IL-1, suggesting that macrophage apoptosis and pyroptosis in IVD in vivo is a significant source of cytokine release.^58^

IL-18, a member of the IL-1 superfamily with structural similarity to IL-1β, is a tightly regulated inflammatory cytokine activated by the intracellular protease caspase-1. IL-18 can enhance the expression of inflammatory mediators such as TNF-α, PGE2, and cyclooxygenase-2 (COX-2).^59^ Zhang et al. revealed that both mRNA and protein expression levels of IL-18 were significantly elevated in the NP tissues of patients and mouse models with IVDD. Silencing IL-18, through Ad-sh-IL-18 treatment, alleviated the progression of IVDD.^60^

IL-6 is an important characteristic and pathogenic factor for IVD degeneration.^53^ Research indicates that IL-6 levels are low within the healthy IVD, but in degenerated IVDs. IL-6 expression is significantly elevated and correlates with the severity of the patient’s back pain.^61,62^ IL-6 activates the JAK/STAT3 pathway, increasing the expression levels of COX-2 and MMP-13, thus contributing to IVDD without involving classical inflammatory factors such as IL-1β or TNF-α.^45,63^ IL-6 can induce macrophages to polarize towards an M1 phenotype, which then enhances the secretion of various inflammatory mediators through autocrine signaling.^64^ Rebecca K. Studer et al. reported that IL-6 can amplify the inflammatory induction effect of IL-1β and TNF-α on human NPCs. This suggests a positive feedback loop among these three cytokines, contributing to a persistent local inflammatory microenvironment and further amplifying the inflammatory response within the IVD.^52^

Pro-inflammatory cytokines can stimulate excessive production of ROS during cellular metabolism, which in turn can initiate oxidative stress (OS), accelerating the progression of IVDD. Oxidative stress is also one of the primary triggers of inflammatory responses in degenerative IVDs. For instance, H_2_O_2_ intervention significantly promoted the expression of IL-1β, IL-6, TNF-α, and inducible nitric oxide synthase (iNOS) in rat NPCs. Additionally, ROS can exacerbate inflammation by inducing mitochondrial dysfunction and promoting NLRP3 activation. This creates a feedback loop where oxidative stress enhances inflammation, which can further aggravate oxidative stress. TNF-α and IL-1β can induce oxidative stress by increasing ROS levels and suppressing superoxide dismutase (SOD) expression in NPCs. This evidence suggests a complex regulatory network between oxidative stress and inflammation that accelerates IVDD progression.^65^ Thus, ROS is regarded as one of the therapeutic targets for IVDD.^66^ High levels of ROS have the capacity to activate M1 macrophages, which are abundant in degenerated IVDs. In turn, activated M1 macrophages further produce ROS and proinflammatory mediators, creating a vicious cycle that exacerbates IVDD^67^ (Fig. 2a).

Li et al. utilized single-cell transcriptome analysis to systematically examine the differences in OS-related functions among various cell populations within degenerative IVD tissues and outlined the longitudinal changes in immune cells, particularly monocytes/macrophages, during IVDD progression. They found that OS-related levels in severely degenerated IVD tissues were significantly higher than in early degenerative tissues, with monocytes/macrophages markedly activated in late-stage degenerative IVD tissues. Moreover, they assessed OS-related functions for each cell subtype, revealing that NPCs and nucleus pulposus progenitor cells had the highest OS-related scores. Among immune cells, monocytes/macrophages were identified as the most correlated immune cells with OS-related functions, indicating that macrophages are key immune cells closely associated with oxidative stress during IVDD progression.^68^ Oxidative stress reactions caused by excessive ROS can further stimulate various abnormal signaling pathways in IVD cells, such as MAPK and NF-κB pathways, ultimately intensifying both local and systemic oxidative stress.^69^

In the inflammatory balance of IVDD, M2 macrophages play a limited but significant anti-inflammatory role. M2 macrophages secrete IL-10 and TGF-β, which are the primary anti-inflammatory factors regulating inflammation in IVDD. IL-10 is an important anti-inflammatory cytokine that functions through a transmembrane receptor complex to regulate various immune cell functions.^70^ Yin et al. developed a rat model of cervical degenerative disease induced by unbalanced dynamic and static forces, and discovered that IL-10 levels sharply increased one month after model induction and were significantly higher than those in the control group three months post-induction.^71^ The increase in IL-10 in IVDD is likely a result of an anti-inflammatory response, which is necessary to prevent excessive stimulation and tissue destruction.^72^ IL-10 can suppress the pro-inflammatory activity of natural killer (NK) cells, monocytes, and macrophages, and decrease the production of pro-inflammatory cytokines.^73,74^ Jun Ge and others found that IL-10 attenuates pro-inflammatory cytokines, antigen presentation, and aberrant immune responses, and enhances immune tolerance. IL-10 increases the degradation of TNF-α by regulating the p38 MAPK pathway.^75^

TGF-β is a multifunctional cytokine and a crucial mediator that regulates cell differentiation, apoptosis, tissue fibrosis, and migration. The TGF-β/Smad signaling pathway is a canonical mechanism for various cellular processes.^76,77^ Studies have suggested that TGF-β signaling may play a significant regulatory role in the process of IVDD.^78–80^ Researchers have confirmed that TGF-βs and TGF-β receptors are highly expressed in human degenerative IVD tissues compared to normal IVD tissues.^81–83^ TGF-β1 has been reported to mitigate inflammation and oxidative stress in IVDD, the involvement of TGF-β1 in inflammation regulation may be linked to the NF-κB pathway.^84,85^ Sun et al. discovered that both knockdown and overexpression of TGF-β1 influenced the activation of the NF-κB pathway.^86,87^ Moreover, Zhang J et al. reported that TGF-β1 could downregulate the expression of CCL4 by activating the MAPK pathway, thereby reducing immune cell infiltration. Furthermore, TGF-β1 and IL-10 can mutually enhance their anti-inflammatory effects.^88^ Therefore, activated TGF-β signaling can alleviate the inflammatory response in the IVD by blocking both the initiation and maintenance of inflammation.^89^

Pro-inflammatory cytokines dominate the inflammatory microenvironment in the IVD. This inflammatory microenvironment causes abnormal cell activities in disc cells, including aberrant proliferation, abnormal PCDs, and senescence. These activities impair the ECM metabolic function of the cells and trigger LBP (Table 2). By deeply studying the regulatory mechanisms of macrophages on the inflammatory microenvironment, we can provide a theoretical foundation for developing novel anti-inflammatory treatment strategies.

**Table 2:** Functions of macrophage related cytokines

Source	Factors	Function		
		Inflammatory regulation	Cellular activities	ECM metabolism
M1	TNF-α	1. Pro-inflammatory cytokines↑^51–53^: IL-6↑, IL-8↑, IL-17↑, TNF-α↑ 2. Chemokines↑^51^: CCL3↑ 3. Pain-related factors↑^51–53^: NO↑, PGE2↑	Proliferation↑^125^ Senescence↑^115–117^ Apoptosis↑^99^ Pyroptosis↑^100^ Ferroptosis↑^106^ Autophagy↓^107^	Catabolism↑^135–137^: MMP-1↑, MMP-3↑, MMP-13↑, ADAMTS-4↑, ADAMTS-5↑, ACAN↓, Col II↓
M1	IL-1β	1. Pro-inflammatory cytokines↑^56^: IL-6↑, IL-8↑, IL-17↑, TNF-α↑ 2. Chemokines↑^51,57^: MCP-1, CCL3↑ 3. Pain-related factors↑^97^: COX-2↑, PGE2↑	Proliferation↓^122^ Senescence↑^115–117^ Apoptosis↑^92–95^ Pyroptosis↑^97^ Ferroptosis↑^106^ Autophagy↓^107^	Catabolism↑^133,134^: MMP-1↑, MMP-3↑, MMP-9↑, MMP-10↑, MMP-13↑, ADAMTS-4↑, ACAN↓, Col II↓
M1	IL-18	1. Pro-inflammatory cytokines↑^59^: TNF-α↑ 2. Pain-related factors↑^59^: COX-2↑, PGE2↑	Apoptosis↑^59^ Pyroptosis↑^59^	Catabolism↑^270^: MMP-13↑, ACAN↓, Col II↓
M1	IL-6	1. Pain-related factors↑^45^: COX-2↑, PGE2↑ 2. Synergistic effect^52^: Enhance pro-inflammatory effects of IL-1β and TNF-α 3. Promote M1Mφ polarization^64^	Ferroptosis↑^105^ Autophagy↓^111^	Catabolism↑^52,138^: MMP-2↑, MMP-13↑, ACAN↓, Col II↓
M2	IL-10	1. Pro-inflammatory cytokines↓^75^: TNF-α↓, IL-1β↓ 2. Synergistic effect^88^: Enhance anti-inflammatory effects of TGF-β		Anabolism↑^75^: ACAN↑, Col II↑, Sox-9↑
M2	TGF-β	1. Pro-inflammatory cytokines↓^87^: TNF-α↓, IL-1β↓ 2. Chemokines↓^88^: CCL4↓ 3. Synergistic effect^72^: Enhance anti-inflammatory effects of IL-10	Proliferation↑^129^ Senescence↓^120^ Apoptosis↓^112,113^ Autophagy↑^107^	Anabolism↑^139^: ACAN↑, Col II↑, MMP-3↓

*M1* M1 macrophage, *M2* M2 macrophage, *TNF-α* tumor necrosis factor α, *IL* interleukin, *COX-2* Cyclooxygenase2, *PGE2* Prostaglandin E2, *NO* nitric oxide, *MCP1* Monocyte Chemoattractant Protein1, *CCL* Chemokine (CC motif) ligand, *ADAMTS* A Disintegrin and Metalloproteinase with Thrombospondin Motifs, *MMP* matrix metallopeptidase, *ACAN* Aggrecan, *Col II* Collagen II, *TGF-β* Transforming Growth Factor β, *Sox-9* SRYBox Transcription Factor 9

### Macrophages regulate cellular activities of IVD cells

Inflammatory microenvironment significantly impacts the cellular activities of IVD cells. Specifically, these mediators can increase abnormal PCD, promote cellular senescence and suppress cell proliferation by activating relevant signal transduction pathways. This ultimately leads to a continuous loss of IVD cells and a decline in cellular functions.

An increase in abnormal PCD activities, particularly apoptosis and pyroptosis under inflammatory conditions, is a significant factor contributing to the loss of IVD cells (Fig. 2b). Apoptosis is a regulated cellular process, intricately designed to maintain cellular homeostasis by removing damaged or unnecessary cells.^90^ Pyroptosis is an inflammatory form of PCD that can cause cell membrane collapse and release inflammatory mediators, including IL-1β and IL-18.^91^

IL-1β-induced apoptosis is a complex process involving multiple signaling pathways, notably the NF-κB, MAPKs, and phosphoinositide 3-kinase (PI3K)/Akt pathways.^92^ In IVDs, IL-1β has been found to mediate apoptosis in IVD cells.^93,94^ IL-1β promotes the production of pro-apoptotic proteins, including cleaved-caspase 3 and Bax, and reduces the production of anti-apoptotic agents in NPCs.^40,95^ NLRs include inflammasome receptors and/or sensors that promote the maturation of caspase 1, IL-1β, IL-18, and gasdermin D, driving inflammation and PCDs.^96^ Ma et al. found in a rat IVD model that IL-1β could induce NLRP3 inflammasome activation and pyroptosis, leading to mitochondrial oxidative stress damage and dysfunction in NPCs.^97^ Xu et al. discovered a vicious cycle of the IL-1β/NLRP3 inflammasome during IVDD, which causes the degradation of the ECM.^98^

TNF-α is a classical apoptotic inducer and activator of the death receptor pathway. Tumor Necrosis Factor Receptor 1 (TNFR1), TLRs, and interleukin-1 receptor (IL-1R) are recognized as damage-associated molecular patterns (DAMPs)/pathogen-associated molecular patterns (PAMPs), which form a receptor-specific multiprotein signaling complex in response to various inflammation-inducing stimuli. This complex plays a crucial role in the apoptosis of IVD cells by controlling the JNK/ERK-MAPK and NF-κB signaling pathways in NPCs during IVDD, upregulating pro-apoptotic proteins and downregulating anti-apoptotic proteins, thereby inducing cell apoptosis.^99^ This complex also triggers the translocation of the NF-κB dimer to the nucleus, resulting in the transcription of NLRP3, pro-IL-1, and pro-IL-18, and triggers pyroptosis.^100^

ROS has been shown to promote apoptosis in NPCs through endoplasmic reticulum stress.^101,102^ Additionally, ROS is closely associated with ferroptosis. Ferroptosis, an iron-dependent form of regulated cell death characterized by the accumulation of lipid ROS, plays a significant role in IVDD.^103^ Dou et al. found that after intervention with conditioned medium (CM) from Lipopolysaccharide (LPS)-stimulated macrophages, both total intracellular iron levels and ROS levels in NPCs significantly increased compared to untreated NPCs. The expressions of ferroptosis repressors GPX4 and SLC7A11 were significantly reduced, while levels of ferroptosis-promoting enzymes such as ACSL4 and LPCAT3 were elevated in CM-treated NPCs, indicating that macrophages can induce ferroptosis in NPCs.^104^ Sheng et al. demonstrated that IL-6 could induce iron overload, lipid peroxidation, and ferroptosis in cartilage endplate cells (CEPCs) from human IVDs.^105^ Given that ferroptosis is closely associated with oxidative stress, IL-1β, IL-18, and TNF-α could potentially promote the ferroptosis process by regulating ROS production.^106^

Autophagy is a cellular process for recycling damaged organelles and proteins, providing energy for cells and removing unwanted cellular structures to prevent damage accumulation.^107^ Basal cell autophagy can delay IVDD and exert a protective effect.^108^ Helen E. Gruber et al. found a significant upregulation of autophagy-related genes in more degenerated IVD compared to healthy IVD.^109^ TNF-α and IL-1β can activate the NF-κB pathway, which has been shown to mutually inhibit autophagy, thereby reducing autophagic activity.^107^ In the study by Yi et al., inhibition of the NF-κB pathway led to an upregulation of the autophagy marker LC3-II, a downregulation of p62, and reduced levels of TNF-α and IL-1. Concurrently, the use of the autophagy inhibitor chloroquine significantly increased the expression of TNF-α and IL-1.^110^ Furthermore, Lin et al. demonstrated that microRNA-21 could enhance the IL-6 inflammatory response and diminish the autophagic capacity of cells.^111^

TGF-β has been reported to reduce apoptosis and cellular senescence.^112,113^ However, in a predominantly pro-inflammatory microenvironment, the effects of TGF-β are limited. These abnormal cell losses and functional declines ultimately result in abnormal ECM metabolism in the intervertebral disc.

Persistent inflammatory stimulation inevitably leads to stress-induced premature senescence (SIPS) (Fig. 2c), one of the major contributors to IVD degeneration.^114^ TNF-α and IL-1β are the primary factors that induce senescence in NPCs, as demonstrated by the upregulation of p16, p53, and SA-β-Gal, which are markers of stress-induced premature aging.^115–117^ Growing evidence demonstrates that cellular senescence is one of the major contributors to IVD degeneration, and TGF-β signaling can regulate cellular senescence in many other cell types.^118,119^ Yokozeki et al. found that TGF-β expression an CD206-positive resident M2 macrophages decreases with age in IVDs.^23^ Duan et al. found that BSHXF-medicated serum can delay the senescence of NPCs by regulating the TGF-β1/Smad pathway, indicating that TGF-β1 is closely involved in the senescence of NPCs.^120^

The proliferation activities of IVD cells can be regulated by macrophage-related factors (Fig. 2d). The MAPK pathway is known to diminish the number of surviving cells in vitro by inhibiting cell proliferation and promoting cellular senescence.^121^ It is considered a primary pathway involved in cell proliferation and survival that is mediated by extracellular stimuli.^41^ Wang et al. found that stimulation with IL-1β significantly suppressed the proliferation of NPCs.^122^ Wu et al. discovered that IL-1β activation of the MAPK/ERK pathway leads to reduced expression of histone deacetylase 4, altered COLII expression, and increased levels of TNF-α, IL-6, and MMP-3. This results in the blockade of the G0/G1 cell cycle phase in NPCs.^123^

It is generally believed that in an inflammatory microenvironment, the proliferation of IVD cells is inhibited. However, some researchers have observed the formation of cell clusters in damaged NP areas, inferring that aberrant proliferation of these cells is the main cause of cell cluster formation.^124^ Another key pro-inflammatory factor, TNF-α, demonstrates contrasting effects in the regulation of IVD cell proliferation activities. TNF-α promotes NP cell proliferation in IVDD through various pathways, including NF-κB, JNK, p38 MAPK, and Notch signaling, highlighting its significant role in the formation of cell clusters characteristic of IVDD.^125^ Chen et al. treated rat NPCs with different concentrations of TNF-α, both in the presence and absence of ER stress inhibitors, and observed that TNF-α stimulus upregulated ER stress markers and initiated the unfolded protein response (UPR), promoting cell proliferation. They further noted that TNF-α induced apoptosis in some NPCs at early stages, while accelerating the proliferation of surviving cells. These effects were reversed by ER stress inhibitors, which reduced cell proliferation and enhanced apoptosis, indicating that TNF-α stimulation promotes NPC proliferation by activating ER stress and initiating the UPR.^126^

Research indicates that TGF-β1 can enhance the proliferation of AFCs and synergistically interact with certain growth factors, such as insulin-like growth factor-I (IGF-I) and fibroblast growth factor-2 (FGF-2), to promote cell proliferation.^127,128^ In rat NPCs, the external application of TGF-β1 facilitates cell cycle progression and stimulates cell proliferation through the regulation of c-Myc signaling and the MAPK pathway.^129^ Hiyama et al. demonstrated that the SMAD signaling pathway could suppress Wnt/β-catenin signaling, thereby sustaining the proliferation of rat NPCs treated with lithium chloride.^130^

### Macrophages regulate ECM metabolism for tissue remodeling

In clinical observations, it has been noted that the herniated part of the IVD in some patients has shrunk or even disappeared. Clinically, this phenomenon where a herniated IVD spontaneously shrinks or disappears without surgical intervention is termed reabsorption.^131^ Researchers believe this may occur as sequestrated discs trigger an inflammatory response characterized by neovascularization and immune cell-mediated degradation, followed by phagocytosis or degradation by immune cells.^11^ Autio et al. reported that the larger the volume of the herniated lumbar disc, the greater the likelihood of spontaneous reabsorption, possibly due to the larger surface area allowing more extensive vascularization and macrophage infiltration.^132^ Reabsorption may be related to macrophage-mediated remodeling activities of IVD cells. Macrophages can achieve remodeling through phagocytosis and regulation of ECM metabolism (Fig. 2e).

Pro-inflammatory cytokines secreted by M1 macrophages can promote the catabolic activities of ECM in IVD cells. IL-1β is known to upregulate the expression of various MMPs, specifically MMP-1, MMP-3, MMP-9, MMP-10, MMP-13, and ADAMTS-4, contributing to the degradation of IVD components, while also decreasing the expression of ACAN and collagen II.^133,134^ Similarly, TNF-α increases the expression of MMP-1, MMP-3, MMP-13, ADAMTS-4, and ADAMTS-5, promoting the breakdown of the ECM and lowering the TIMP-1/MMP-3 ratio, thereby exacerbating the inflammatory and degenerative metabolism of the disc.^135–137^ IL-6, through the trans-signaling pathway, inhibits the production of collagen II and proteoglycans by NPCs and induces the secretion of MMP-2, MMP-13, COX-2, and PGE2, accelerating ECM degradation.^52,138^

M2c macrophages, identified by the cell surface marker CD163, are known for their remodeling phenotype as they secrete high levels of MMP-7 and MMP-8.^36^ It has also been reported that exosomes from M2c macrophages play a crucial role in mediating rebalancing effects on ECM metabolism, effectively promoting ECM synthesis while inhibiting its degradation. Exosomes from M2c macrophages enhance the activity of the TGF-β/Smad3 pathway in NPCs, promoting the synthesis of essential ECM components such as aggrecan and collagen II, which are vital for the structural integrity and function of the IVD.^139^ This underscores the significant role of M2c macrophages in IVD remodeling.

The progression of IVD remodeling is accompanied by fibrosis of NP tissue. Swahn et al. conducted single-cell sequencing on healthy and degenerative IVDs, discovering an increase in several fibrotic clusters within degenerative IVDs, suggesting that IVDD is associated with tissue remodeling. In NP and AF cells, thrombospondin protein promoted the expression of genes linked to TGFβ/fibrosis signaling.^140^

Macrophage infiltration may also play a role in NP fibrosis. Current studies demonstrate that the overexpression of CEMIP in degenerated IVD tissues facilitates NP fibrosis and neovascularization induced by macrophage infiltration. Silencing the fibrosis marker CEMIP can reverse the fibrotic phenotype of NPCs.^141^ Both M1 and M2 macrophages can promote the progression of NP tissue fibrosis. M1 macrophages strongly stimulate IVD inflammation and are identified as key mediators of NP fibrosis, while M2 macrophages exert profibrotic effects across various diseases.^142–144^

IL-1β has been shown to stimulate the expression of collagen types I and III in human NPCs, indicating a fibroblast-like phenotype.^145^ Hou et al. demonstrated that NF-κB activation is crucial in the production of myofibroblasts in inflammatory lung fibrosis.^146^ Moreover, the NLRP3 inflammasome is associated with inflammation, pyroptosis, ECM degradation, and cell apoptosis.^47^ The NF-κB–NLRP3–Caspase-1–IL-1β–IL-18 axis has been shown to create a pathological loop controlling cardiac fibrosis alongside the TGF-β/Smad signaling pathway.^147^

The TGF-β/Smad signaling pathway plays a critical role in the fibrotic process.^148^ TGF-β signaling aids IVD repair by promoting ECM synthesis, reducing catabolic activity, and suppressing inflammation.^149–152^ TGF-β can partially reverse the upregulation of matrix-degrading enzymes induced by pro-inflammatory cytokines by modulating the MAPK and NF-κB pathways. Numerous studies have shown that TGF-β treatment not only suppresses the release of IL-1β and TNF-α but also inhibits the increased expression of MMPs induced by inflammatory cytokines.^87,88,153,154^ For instance, injection of fibroblasts into the disc induces a fibrotic phenotype in NPCs through TGF-β secretion, resulting in effective disc height maintenance, slowed endplate degeneration, improved MRI signals, and enhanced overall tissue structure.^155^ However, excessive and sustained TGF-β activation may lead to IVDD, and inhibiting its aberrant activity may slow IVDD progression.^78,148^ Numerous studies show that dysregulation of TGF-β1/Smad is an important pathogenic pathway in tissue fibrosis across various organs, including the lungs, liver, and kidneys.^156,157^ Rapid collagen deposition induced by TGF-β stimulation is thought to contribute to tissue strength in multi-organ fibrosis.^158^

Research suggests that NPCs contribute to fibroblast differentiation through the TGF-β receptor 1 (TGF-βR1)-Smad2/3 pathway, inflammation activation, and mechanosensitive mechanisms. NP fibrosis is also associated with abnormal MMP activity, aligning with the role of matrix metalloproteinases in tissue fibrosis regulation. Activated macrophages additionally produce pro-fibrogenic factors, such as TGF-β1, enhancing the proliferation and activation of collagen-producing fibroblasts. The finding of colocalization between the macrophage marker MMP-12 and α-smooth muscle actin (α-SMA) in the NP of induced IVDD appears to supports this transition.^159^

In summary, as a protective mechanism of the immune system, macrophages exert a dual effect on ECM metabolism. On one hand, to eliminate protruded IVD, macrophages promote the self-absorption of the protruded NP, and alleviate symptoms. On the other hand, long-term interventions dominated by catabolic metabolism in a pro-inflammatory environment will inevitably exacerbate the progression of IVDD, especially after macrophages invade the interior of the IVD. This prolonged catabolic stimulus will lead to the disintegration of the natural ECM components of the IVD. Although factors like TGF-β can mitigate catabolic metabolism and repair the damaged IVD, the ECM of the IVD cannot return to a healthy state, and over time, the fibrotic NP tissue will be unable to fulfill its function.

### Macrophages regulate discogenic LBP

Discogenic LBP is a distinct category of back pain originating from the IVD, characterized by magnetic resonance imaging (MRI) findings that show structural alterations at lumbar levels, predominantly involving nociceptive and neuropathic pain.^160^ As an internally avascular and aneural structure, discogenic LBP is closely related to the progression of IVDD. Discogenic LBP results from nerve innervation and sensitization, where nerves are typically distributed in the outer region of the AF. Under the influence of IVDD, nerve growth can extend into the inner annulus and NP, likely contributing directly to pain generation.^161^ Peng et al. collected lumbar IVD specimens from patients with discogenic LBP and performed histological examinations. They found abundant macrophages in the granulation tissue zone of painful IVDs, indicating a close correlation between macrophages and discogenic LBP (Fig. 2f).^162^

During the process of nerve invasion, nerve growth factor (NGF) plays a crucial role in chronic discogenic pain by sensitizing disc-innervating neurons and promoting nerve ingrowth into degenerated discs, a primary factor in pain generation. This is supported by findings that show significantly higher NGF levels in herniated discs compared to non-herniated discs.^163,164^ M1 macrophages create a chronic inflammatory microenvironment by releasing pro-inflammatory cytokines and NGF, inducing nerve growth into the inner AF.^165^ Yang et al. encapsulated MnO2 nanoparticles with macrophage cell membranes overexpressing TrkA, enabling specific targeting of macrophages, reducing M1 macrophage polarization, and decreasing NGF expression, thereby alleviating pain.^166^

Macrophages in injured IVDs may contribute to neurite and angiogenesis by releasing pro-inflammatory factors.^167^ A systematic review revealed that M1-related cytokines, such as high levels of TNF-α, TNFR1, IL-6, IL-8, and IFN-γ, were all associated with high visual analog scale (VAS) scores. Conversely, high levels of M2-related cytokines, including IL-4 and IL-10, were associated with lower VAS scores.^168,169^ Inflammatory cytokines like TNF-α and IL-1β promote tissue destruction and enhance the sensitivity of nerve fibers, thereby triggering pain.^170^ Lee et al. observed macrophage infiltration as early as 4 days post-injury in mice, which persisted at subsequent time points, particularly notable at 12 months on the dorsal aspect of the IVDs. The density of nerve fibers, measured using PGP9.5 and calcitonin gene-related peptide (CGRP) immunoreactivity, also showed an increase in the injured discs. The increase in macrophages and nerve fibers in the dorsal aspect of the IVDs is likely related to more extensive physical disruptions such as protrusions and herniations. The researchers also found that macrophages, by secreting inflammatory mediators (such as IL-1β and TNF-α), stimulate IVD cells and promote the release of NGF. This indirectly promotes nerve fiber growth and pathological neural activity, thereby intensifying pain perception.^171^

Miyagi et al. discovered that TNF-α and IL-1β levels significantly decreased with macrophage depletion, while NGF and VEGF levels increased in injured IVDs irrespective of macrophage presence. TNF-α treatment upregulated NGF, VEGF, COX-2, and mPGES1 in F4/80-negative disc cells, suggesting that macrophages modulate pain through these inflammatory cytokines. During IVDD, the loss of nuclear material and tears in the AF might directly trigger pain receptors, causing discogenic LBP.^172^ Thus macrophage involvement in the remodeling process of IVDs may trigger pain receptors, leading to discogenic pain.

In summary, macrophages play a role throughout the entire process of IVDD, from its onset and progression to dysfunction. By understanding how macrophages infiltrate and participate in each pathological event of IVDD, we can better comprehend their roles in these events. This knowledge allows us to develop targeted interventions at different stages of IVDD, providing evidence and a theoretical foundation for clinical translation.

## Involvement of macrophages in endplate degeneration

**Fig. 3:**
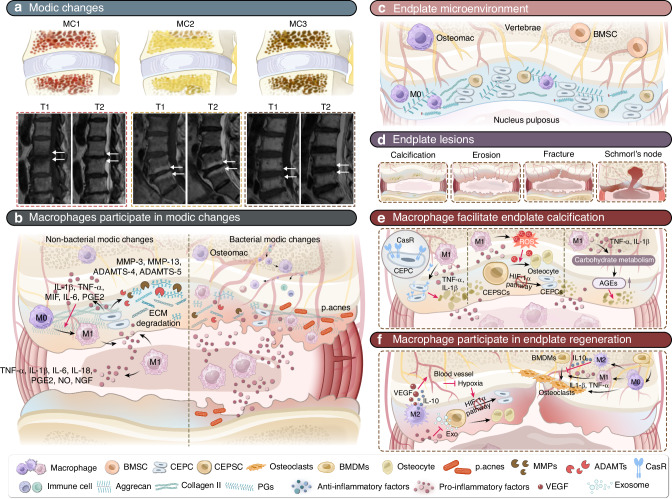
Role of macrophages in endplate degeneration. **a** Classification and MRI characteristics of Modic changes. MC1 (low T1 and high T2 signals) is associated with edema and inflammation. MC2 (high T1 and T2 signals) indicates a progression to fatty involution. MC3 (low T1 and T2 signals) signify vertebral endplate bone sclerosis. **b** Macrophages participate in MCs. In non-bacterial MCs, macrophages accelerate endplate degeneration by promoting the expressions of pro-inflammatory cytokines, MMPs, and ADAMTs. In bacterial MCs, a wider variety of tissue-derived macrophages, including peripheral blood macrophages and osteal macrophages, contribute to endplate inflammation, leading to more severe endplate degeneration. **c** Schematic of a healthy endplate microenvironment. CEPCs synthesize an extracellular matrix primarily composed of collagen II and ACAN. CEPSCs are stem cells for intrinsic repair of the endplate. **d** Schematic of endplate lesions. Endplate lesions includes calcification, erosion, fractures, and Schmorl’s nodes. **e** Macrophages promote endplate calcification. Macrophages enhance CASR expression in CEPCs and regulate carbohydrate metabolism to increase AGEs expression by releasing pro-inflammatory cytokines, thereby promoting calcification. Additionally, macrophages promote calcification by increasing ROS production, which induces osteogenic differentiation of CEPSCs. **f** Macrophages in endplate regeneration. Macrophages promote neovascularization of the endplate by releasing VEGF. Macrophages participate in bone remodeling by regulating osteoclast formation and bone resorption activity

Distinguished from LBP caused by AF rupture, endplate degeneration is another significant cause of LBP. The endplate, as a junctional tissue between the vertebra and NP, possessing distinct mechanical and metabolic microenvironment characteristics. The endplate is rich in sensory nerve terminals, and the cartilage canal and vascular buds in the vertebral endplate are specific fine structures.^173^ Anatomically, the endplate can be divided into the bony endplate and the cartilaginous endplate. The bony endplate connects with the vertebra, while the cartilaginous endplate is a thin layer of hyaline cartilage that connects with the nucleus pulposus. The blood supply ends in the CEP, making the CEP a critical transportation junction (Fig. 3c). The CEP plays a crucial role in nutrition and mechanical stability within the IVD.^174^

Endplate degeneration can lead to metabolic disturbances and mechanical dysfunction within the IVD. Furthermore, the degenerated CEP can act as a potential source of inflammatory mediators, such as TNF-α, IL-1β, IL-6, and macrophage migration inhibitor factor (MIF), which contribute to intervertebral disc degeneration.^175^ On the other hand, a single-cell sequencing study reported the presence of M0 macrophages in healthy and degenerative CEP tissues.^176^ As the endplate is a partially vascularized tissue connected to the circulatory system and adjacent to the vertebra, macrophages from the circulatory system and bone marrow likely play a role in the process of endplate degeneration.

Currently, most discussions about IVDD revolve around the microenvironment and cellular interaction mechanisms between the nucleus pulposus and annulus fibrosus, as well as the outcomes of IVDD, such as disc herniation and discogenic back pain. Mechanistic analysis of endplate degeneration is relatively rare. Modic changes (MCs) and endplate lesions are the two main research directions concerning endplate degeneration. We will explore the role of macrophages in endplate degeneration from these two perspectives.

### Macrophage-related inflammatory responses involved in MCs

MCs are pathological changes of vertebral endplates and adjacent bone marrow, detectable through MRI.^177^ Based on MRI characteristics, MCs are classified into three types. MC1 (low T1 and high T2 signals on MRI) is associated with edema and inflammation. MC2 (high T1 and T2 signals) indicates a progression to fatty involution. MC3 (low T1 and T2 signals) signifies vertebral endplate bone sclerosis (Fig. 3a).^178^ These three types represent different manifestations of endplate degeneration on MRI and can exist individually or in combination.^179^

MCs are closely associated with inflammation. In the CEP, macrophage-related inflammatory factors IL-1β and TNF-α create an inflammatory microenvironment that upregulates the expression of MMP-3, MMP-13, ADAMTS-4, and ADAMTS-5. These enzymes are critical for the degradation of the cartilage ECM and induce abnormal PCDs, which promote the degeneration of CEPCs (Fig. 3b).^180,181^ Huang et al. found that macrophages play a key role in the degeneration of the CEP and the progression of IVDD. The use of 4-Octyl Itaconate (4OI) can significantly inhibit the inflammatory response induced by LPS in RAW264.7 cells, reducing the secretion of pro-inflammatory factors such as IL-1β and TNF-α, thereby alleviating the degeneration of rat CEP cells induced by the supernatant from RAW 264.7 cells.^182^ Aboushaala et al. found several statistically significant blood biomarkers in patients with MC, including elevated levels of CCL5 and significantly reduced MIF.^183^ In degenerated endplates with MC, the expression of MIF and its receptor CD74 was significantly increased in CEP cells.^184^ This suggests that immune cells from the circulatory system are mobilized to the endplate to initiate and maintain the immune response.

MIF is a pro-inflammatory cytokine, named for its ability to inhibit the random migration of macrophages. It is an immunomodulator that is rapidly released from both immune and non-immune cells in response to various stimuli.^185^ MIF plays a role in the activation of macrophages, stimulating them to produce other pro-inflammatory cytokines such as TNF-α, IL-1β, and IL-6, which amplify the inflammatory response. MIF also enhances the production of ROS and NO, which are crucial for pathogen killing. In chronic inflammatory diseases, MIF contributes to the persistence of macrophage activation, leading to tissue damage.^186^ Xiong et al. found that LPS and TNF-α dose-dependently upregulate MIF expression in CEPCs. Meanwhile, MIF dose-dependently increases the secretion of IL-6, IL-8, and PGE2 in CEPCs. The simultaneous application of the MIF antagonist ISO-1 can reduce the secretion of these cytokines,^184^ indicating that MIF plays an important role in endplate inflammation. Gjefsen et al. found that, by analyzing the serum levels of 40 cytokines from LBP patients with MCs, six cytokines, including MIF and IL-6, were moderately correlated with pain. Principal component analyses revealed clustering and separation of patients with LBP and controls, indicating 10 cytokines contributing to the separation. MIF alone accounted for 92% of the total contribution. This suggests that inflammation caused by the sustained activation of macrophages by MIF may be the main cause of LBP in patients with MCs.^187^

In recent years, researchers have discovered that the immune response triggered by Propionibacterium acnes (P. acnes) may be an important cause of MCs. Dudli et al. aseptically isolated P. acnes from a symptomatic human L4/5 disc with MC1 and injected it into rat tail discs. They found that after inoculation, P. acnes could proliferate within the disc, induce degeneration, and cause changes similar to MC1.^188^ Another study demonstrated that the injection of P. acnes into rabbit lumbar IVDs resulted in Modic changes over time, indicating that this bacterium can induce degenerative changes in the endplates.^189^

Heggli et al. suggested that MCs may have bacterial and non-bacterial subtypes. They collected disc samples adjacent to MC1 and control vertebra samples from patients undergoing spinal fusion surgery. Using 16S qPCR, they quantified the genomic copy numbers (GCNs) of P. acnes and employed RNA sequencing to assess the transcriptome characteristics of MC1 and control group bone marrow to differentiate the immune cell characteristics of the two groups. They evaluated the relationship between P. acnes genomic copy number and plasma cytokines through correlation analysis. They found that MC1 patients with “high” P. acnes GCNs exhibited innate immune cell characteristics (neutrophils, macrophages/monocytes) in bone marrow and upregulated pro-inflammatory cytokines related to neutrophil and macrophage/monocyte functions, consistent with host defense against bacteria. Conversely, MC1 patients with “low” P. acnes GCNs exhibited increased adaptive immune cell characteristics (T cells and B cells) in bone marrow and elevated IL-13 levels in plasma.^190^ In a rat model, Zhang et al. found that applying P. acnes significantly induced CEP degeneration and upregulated MIF expression. They discovered that P. acnes induced MIF expression in a concentration-dependent manner. MIF then upregulated the expression of MMP-13 and promoted the secretion of IL-6 and IL-1β.^191^

Wang et al. further used P. acnes to induce MCs in a rat model and found that osteal tissue macrophages increased in the endplate subchondral bone after inducing MCs. They confirmed that osteal tissue macrophages promote endplate osteosclerosis through the OSM-STAT3/YAP1 signaling axis.^192^ These findings suggest that the macrophage-related inflammatory response caused by P. acnes infection may be a primary pathogenic factor for MCs (Fig. 3b).

### Macrophage involvement in endplate injury and regeneration

Another research direction for endplate degeneration is endplate lesions. Previously, four types of endplate lesions were identified in cadaveric spines: calcifications, erosions, fractures, and Schmorl’s nodes (Fig. 3d).^193^

Calcification may be the primary cause and initiating factor of endplate lesions and endplate sclerosis. Zehra et al. suggested that the calcified areas near fissures in degenerative cartilaginous endplate tissues are nucleation points for cracks and fissures, and are associated with increased IVD degeneration.^194^

AGEs (advanced glycation end-products) formed under pro-inflammatory conditions are believed to be closely related to IVD calcification. Calcification and endochondral ossification induced by AGEs may be important reasons for endplate sclerosis.^195^ TNF and IL-1β have been reported to promote vascular calcification by stimulating the release of BMP2.^196^ In a diabetic mouse model, researchers found that the deposition of fissures and granular structures in the IVD was associated with AGE accumulation and the expression of the pro-inflammatory cytokine TNF (Fig. 3e).^197^ During inflammation, macrophage-related factors can also trigger microcalcification; TNF-α increases intracellular ROS levels, causing cellular senescence and osteogenic differentiation in cartilaginous endplate stem cells (CESCs) (Fig. 3e).^198^ Meanwhile, pro-inflammatory cytokines such as IL-1β and TNF can participate in disc calcification by increasing the expression of calcium-sensing receptor (CasR) (Fig. 3e).^194,199^ Quan et al. used 4-week-old mice for diabetes modeling and found significant endplate calcification and abnormal chondrocyte appearance in the diabetes group after 6 months. Immunofluorescence showed significantly increased expression levels of CD31 (vascular endothelial marker) and F4/80 (monocyte/macrophage marker) in the diabetes group compared to the control group, with an increased number of F4/80^+^/CD16/32^+^ cells. Transmission electron microscopy observations of the endplate morphology revealed monocyte/macrophage accumulation in the endplate of the diabetes group, accompanied by increased vascular density, distorted collagen fibers, and abnormal chondrocytes. This indicates that under the chronic inflammatory environment of diabetes, increased macrophages and vascularization caused endplate calcification.^200^ This suggests that macrophages and related inflammatory factors may be the cause of endplate calcification and further injury.

Endplate sclerosis is considered a marker of severe endplate degeneration and can occur in any type of MCs endplate, with sclerosis being most severe in type III MCs endplates.^201^ Macrophages participating in endochondral ossification and bone remodeling after endplate injury may be a crucial reason for endplate sclerosis. Abnormal stress can cause microfractures in the endplate, leading to MC and IVDD.^202,203^ Lai et al. found that endplate injury increased CD68 macrophages in the endplate and vertebrae, leading to spinal sensitization and pain. This indicates that macrophages are involved in inflammation after endplate injury and contribute to pain generation.^204^

On the other hand, there are CESCs in the endplate, which serve as an endogenous repair mechanism. CESCs can alleviate IVDD by secreting exosomes, but under a degenerative inflammatory environment, the anti-degenerative efficacy of these exosomes is significantly reduced.^205^ Additionally, CESCs can activate the HIF-1α-related pathway to differentiate into cartilage and repair damaged areas under hypoxic conditions. When hypoxic conditions are disrupted, CESCs tend to undergo osteogenic differentiation, leading to changes in endplate stress distribution and increased susceptibility to other injuries. Neovascularization can disrupt the hypoxic microenvironment, inhibit the HIF-1α-related pathway, and cause CESCs to differentiate into bone rather than cartilage. This osteogenic endogenous repair process may be a key factor in the gradual ossification and sclerosis of the endplate with age and degeneration (Fig. 3f).^206,207^

Through histological analysis of human cartilaginous endplates, Huang et al. found microdamage in the CEPs of 40% of patients with suspected discogenic LBP. Neovascularization and nerve ingrowth occurred in the damaged and proteoglycan-depleted areas of the cartilaginous endplates.^208^ Macrophages have been shown to be involved in the process of neurovascularization. Fan et al., in in vivo and in vitro studies, confirmed that aging macrophages increase pSTAT3 expression in endothelial cells by releasing IL-10. This leads to pSTAT3 directly binding to the promoters of VEGF, MMP2, and PDGF, encouraging their production and resulting in endplate angiogenesis and sclerosis (Fig. 3f).^18^ Macrophages not only contribute to vascularization in aging endplates but also participate in the repair phase of endplate injury. Yamamoto et al. used GFP-labeled bone marrow chimeric rats to create a model of disc injury and degeneration, finding that bone marrow-derived macrophages gradually increased in the endplate and their polarity became M2 dominant as IVDD progressed.^16^ This finding indicates that bone marrow-derived macrophages migrate to the injury area after endplate injury and gradually polarize to the M2 phenotype, exerting anti-inflammatory and pro-repair effects. As mentioned earlier, M2 macrophages can secrete VEGF, which may be an important cause of vascular buds and a significant factor leading to the osteogenic differentiation of CESCs. Therefore, the gradual ossification of the endplate is considered an important marker of increased endplate degeneration.

Abnormal bone remodeling can disrupt the structural integrity of the endplate, leading to impaired nutrient supply to the intervertebral disc, increased expression of catabolic factors, and decreased levels of collagen II and ACAN.^209^ Research indicates that in the osteoporotic microenvironment where the functions of osteoclasts and osteoblasts are imbalanced, the cartilage endplate is more prone to damage, resulting in intervertebral disc degeneration. This suggests that osteoclasts and osteoblasts play crucial roles in the remodeling process of the endplate.^210,211^ The degenerated endplate has increased porosity because of osteoclastic remodeling activity and may be a source of LBP due to aberrant sensory innervation within the pores.^212,213^

Ni et al. confirmed that the number of TRAP^+^ osteoclasts in the endplate significantly increased two weeks after lumbar spine instability (LSI) surgery in mice and remained at a high level until eight weeks post-surgery. Bone resorption in the sclerotic endplate created large bone marrow cavities. This process promoted a significant increase in CGRP^+^ nerve fibers (a marker of pain nerve fibers), which persisted until eight weeks, thereby inducing pain.^214^

Osteoclasts are multinucleated cells derived from bone marrow-derived monocytes (BMDMs), which also give rise to various types of tissue macrophages.^215^ Macrophages can interact with osteoclasts and regulate bone metabolism by secreting various cytokines, and they can also form osteoclasts through fusion and multinucleation to promote bone resorption (Fig. 3f).^216,217^ The genetic or pharmacological clearance of macrophages in LSI surgery mice or aged mice could decrease the number of Trap^+^ osteoclasts in the endplates.^18^ Therefore, during endplate degeneration, macrophages can participate in bone remodeling of the endplate by affecting osteoclast formation.

Macrophages migrate to the injury site during the initial inflammatory phase of a fracture and are present at all stages of fracture healing. They are believed to have a significant impact on osteogenesis.^218^ Numerous studies support that macrophages can promote the osteoblastic differentiation of bone marrow stromal cells and the proliferation, differentiation, and survival of osteoblasts.^219^ Batoon et al. selectively depleted CD169^+^ macrophages and found that bone repair was impaired in both intramembranous and endochondral ossification models. The depletion of CD169^+^ macrophages did not affect osteoclast frequency in either model. This indicates that CD169^+^ macrophages can provide critical anabolic support to osteoblasts independently of osteoclasts during bone homeostasis and repair.^220^

As mentioned, the role of macrophages in endplate degeneration is complex due to the endplate’s connection to external tissues. Macrophages with different polarization states, tissue origins, and subtypes are involved in the process of endplate degeneration. Macrophages play a regulatory role not only in the inflammation and degeneration of the endplate but also in the repair and bone remodeling processes following endplate lesions. Currently, the role of specific subtypes such as CD169^+^ macrophages in osteogenic repair has been revealed, but there is still a lack of relevant research in the context of endplate degeneration. How exactly macrophages participate in the repair process after endplate injury, what roles different subpopulations play during the repair stage, and what relationships exist between macrophages and other immune cells still require further research to uncover.

## Therapeutic strategies for IVDD through regulation of macrophages

Given the pivotal role of macrophages in the degeneration of IVDs, researchers are investigating various therapeutic approaches to mediate macrophages. These include the use of chemical drugs, genetic engineering, exosomes, and tissue engineering strategies. These approaches aim to regulate or alter the behavior and polarization of macrophages to potentially mitigate or reverse the impacts of disc degeneration (Table 3).

**Table 3:** Therapeutic strategies for IVDD through regulation of macrophages

Strategies	Methods	Mechanism	Outcomes	References
Pharmacology	CCR1/2 Antagonists	Inhibit the migration of macrophages	Prevent inflammation and alleviate IVDD	^221^
Pharmacology	17-AAG	Inhibit HSP90, reduce sensitivity to LPS	Inhibit M1 polarization and alleviate IVDD	^142^
Pharmacology	MLT	Modulate SIRT1/Notch pathway	Inhibit M1 polarization, reduce ferroptosis and alleviate IVDD	^226^
Pharmacology	Cordycepin	Inhibit NF-κB pathway	Reduce early inflammation and alleviate IVDD	^227^
Pharmacology	MAG	Downregulate HMGB1/MyD88/NF-κB pathway and NLRP3 inflammasome	Inhibit M1 polarization, reduce inflammation, and alleviate IVDD	^228^
Extracellular Vesicle	PRP-EVs	Regulate NF-κB and MAPK pathways	Inhibit M1 polarization, promote M2 polarization and alleviate IVDD	^231^
Extracellular Vesicle	BMSC-EVs (miR-129-5p)	Inhibit LRG1-dependent p38/MAPK activation	Inhibit macrophage apoptosis and M1 polarization and alleviate IVDD	^233^
Extracellular Vesicle	BMSC-EVs (lncRNA CAHM)	Deliver lncRNA CAHM	Inhibit M1 polarization and alleviate IVDD	^234^
Extracellular Vesicle	dNPC-EVs (miR-27a-3p)	PPARγ/NFκB/PI3K/AKT	Induce M1 polarization of macrophages and exacerbate IVDD	^22^
Gene Regulation	DNMT1 Inhibition (shRNA)	Induce M2 polarization, reduce apoptosis	Enhance M2 polarization, reduce cell apoptosis in degenerated areas, alleviate pain	^237^
Tissue engineering	PC20-PEI600 + TrkA-IN-1	Capture DNA and encapsulate TrkA-IN-1	Inhibit M1 polarization, reduce inflammation and alleviate IVDD	^165^
Tissue engineering	ROS-scavenging scaffold + rapamycin	Load rapamycin, inhibit mTORC1	Promote M2 polarization, alleviate inflammation and IVDD	^238^
Tissue engineering	DexMA hydrogel + fucoidan	Modulate hydrogel mechanical strength and structure	Regulation of macrophage polarization to M2, alleviate inflammation and IVDD	^239^
Tissue engineering	OPF/SMA+IL-4-KGN-PLGA hydrogel	Sequential release of IL-4 and KGN	Promote M2 polarization, alleviate inflammation and IVDD	^240^

*CCR1/2* CC Chemokine Receptor 1& 2, *HSP90* Heat-shock protein 90, *MLT* Melatonin, *SIRT1* Silent mating type information regulation 2 homolog-1, *NF-κB* Nuclear factor kappa-B, *MAG* Magnoflorine, *HMGB1* High mobility group box protein 1, *MyD88* Myeloid differentiation factor 88, *PRP-EVs* Platelet-rich plasma-derived extracellular vesicles, *BMSC-EVs* bone mesenchymal stem cell-derived extracellular vesicles, *miR* microRNA, *MAPK* mitogen-activated protein kinase, *lncRNA* long non-coding RNA, *dNPC-EVs* Degeneration nucleus pulposus cell-derived extracellular vesicles, *PPAR* Peroxisome proliferator-activated receptor, *PI3K* Phosphatidylinositol 3-kinase, *TrkA-IN-1* TrkA-IN-1-cationic nanoparticles-decellularized annulus fibrosus matrix, *mTORC1* Mammalian target of rapamycin complex 1, *OPF/SMA* Oligo [poly (ethylene glycol) fumarate]/sodium methacrylate, *IL-4-KGN* Interleukin 4-kartogenin, *PLGA* poly lactic-co-glycolic acid

### Pharmacological strategies

In the study of IVDD, pharmacological strategies aim to alleviate the condition by modulating macrophage infiltration and the inflammatory response triggered by these cells.

Inflammatory chemokines facilitate the migration of macrophages and neutrophils to damaged or infected tissues, responding to specific chemokine ligands. Preclinical studies by Chou et al. demonstrated that antagonists targeting C-C chemokine receptor 1/2 (CCR1/2) can inhibit the migration of macrophages to the IVD, thereby preventing inflammation and its subsequent induction of LBP. The intradiscal injection of these antagonists effectively alleviates disc inflammation at an early stage, with the CCR1 antagonist particularly showing long-term anti-inflammatory effects.^221^

Promoting the degradation of Heat Shock Protein 90 (HSP90) to inhibit inflammatory signaling cascades has been recognized as a promising approach for treating various inflammatory diseases.^222,223^ Zhang et al. utilized 17-Allylamino-17-demethoxygeldanamycin (17-AAG), an HSP90 inhibitor, to reduce the sensitivity of macrophages to LPS. Simultaneously, 17-AAG also inhibits the polarization of M1 macrophages and dose-dependently restores the inflammatory and metabolic state of NPCs.^142^

Melatonin (MLT) can induce a phenotypic shift from pro-inflammatory M1 macrophages to anti-inflammatory M2 macrophages by binding to its nuclear receptor, ROR (Retinoid-related Orphan Receptor). This binding results in the downregulation of inflammatory cytokines and oxidative stress molecules, contributing to its anti-inflammatory properties.^224,225^ Dou et al. showed that MLT inhibits M1 macrophage polarization by modulating the SIRT1/Notch pathway. Meanwhile, it upregulates GPX4 and SLC7A11, and downregulates ACSL4 and LPCAT3, reducing cellular iron levels and inhibiting ferroptosis in NPCs, thereby alleviating inflammation-induced damage.^226^

Cordycepin is noted for its anti-inflammatory effects. Li et al. demonstrated that cordycepin can reverse the upregulation of the CCL2 gene induced by LPS in NPCs and inhibit macrophage migration, thus reducing early inflammation in these cells. This effect is likely achieved by inhibiting the phosphorylation of IκBα and p65 in the NF-κB pathway stimulated by LPS.^227^ Similarly, magnoflorine (MAG) exhibits comparable anti-inflammatory properties. Zhao et al. reported that MAG inhibits M1 macrophage polarization and reduces inflammation by downregulating the HMGB1/MyD88/NF-κB pathway and the NLRP3 inflammasome, thereby mitigating damage to NPCs in IVDD.^228^

The advantage of pharmacological strategies lies in their rapid action, producing noticeable effects in a short time. These approaches can target specific mechanisms to modulate inflammation and improve intervertebral disc degeneration (IVDD), with a wide range of available drugs offering broad indications. However, the complex mechanisms of these drugs often affect multiple biological pathways, making it challenging to address the intricate degenerative microenvironment of the IVD. Additionally, side effects or adverse reactions may arise.

### Extracellular vesicle and gene regulation strategies

Extracellular vesicles (EVs) encase various bioactive substances crucial for cell communication and can modulate the inflammatory microenvironment, influencing the progression of IVDD.^229,230^ Qian et al. highlighted the therapeutic potential of EVs derived from Platelet-Rich Plasma (PRP). These EVs inhibit the polarization of M1 macrophages by regulating the NF-κB and MAPK pathways and promote the polarization of M2 macrophages through the phosphorylation of STAT6, effectively hindering the pathological progression of IVDD in rats.^231^

EVs derived from bone marrow stem cells (BMSC-EVs) are considered promising therapeutic agents due to their ability to regulate NP cell vitality, ECM synthesis, and apoptosis.^232^ Cui et al.‘s study demonstrates that BMSC-EVs, through the delivery of miR-129-5p, inhibit macrophage apoptosis, M1 polarization, and ECM degradation in NPCs by disrupting LRG1-dependent p38/MAPK activation. This mechanism effectively delays the progression of IVDD.^233^ Li et al.‘s research highlights the role of long non-coding RNAs (lncRNAs) in macrophage polarization, showing that BMSC-EVs significantly inhibit M1 polarization and alleviate IVDD by delivering lncRNA CAHM.^234^

Therapeutic effects can also be achieved by blocking the delivery of EVs that facilitate M1 macrophage polarization.^235^ Zhao et al. discovered that EVs from degenerated NPC (dNPC-EVs) can induce M1 macrophage polarization and exacerbate IVDD by delivering miR-27a-3p, which targets the PPARγ/NFκB/PI3K/AKT signaling pathway.^22^

Gene regulation can also achieve therapeutic effects by intervening in macrophage polarization. Previous studies have linked DNA methyltransferase 1 (DNMT1) to chronic inflammatory diseases and macrophage polarization.^236^ Hou et al. demonstrated the potential of DNMT1 inhibition in inducing M2 polarization of macrophages, enhancing cellular vitality, and reducing cellular senescence and apoptosis, which are beneficial for treating IVDD. They employed short hairpin RNA (shRNA) targeted against DNMT1, delivered via an adeno-associated virus (AAV), to specifically target local macrophages in a lumbar disc degeneration (LDD) mouse model. Their findings revealed that shDNMT1 significantly enhances the ratio of CD206^+^ M2 macrophages to CD86^+^ M1 macrophages, thus promoting M2 cell polarization. This intervention significantly reduced cell apoptosis in the degenerative disc areas of the mice and alleviated the associated pain.^237^

EVs have functions similar to their parent cells and can effectively enhance IVDD by transferring specific RNA or proteins to regulate intercellular communication. They are relatively safe and have few side effects. However, a significant challenge in treating IVDD with EVs is their delivery approach. Direct injection of EVs into the disc leads to a transient release, which may not provide sustained effects, thereby limiting efficacy. Repeated injections could damage the AF and exacerbate degeneration. Therefore, using EVs for IVDD treatment requires a drug delivery platform capable of stable, long-term EV release. Gene regulation strategies offer the advantage of precise control over gene expression with long-lasting effects. However, low gene transduction efficiency may impact therapeutic outcomes, and safety and long-term effects still require further investigation.

### Tissue engineering strategies

In the process of IVDD, macrophages are continuously involved. Pharmacological strategies, extracellular vesicle strategies, and gene regulation strategies often require repeate intradiscal injections, which are difficult to achieve long-term efficacy and can repeatedly damage the AF. Tissue engineering strategies can utilize scaffolds as platforms to carry cells or factors for in situ repair of IVDD. Customized tissue engineering strategies can simultaneously modulate the inflammatory microenvironment and promote tissue regeneration, achieving multiple therapeutic effects.

To regulate the inflammatory microenvironment, Peng and colleagues developed a multifunctional material known as cationic amphiphilic polycarbonate micelles (PC20-PEI600), designed to capture negatively charged DNA through electrostatic interactions and encapsulate hydrophobic drugs. Their study involved using these micelles, loaded with TrkA-IN-1 (TI-cNP-DAF), to effectively inhibit the M1 polarization of macrophages triggered by mitochondria-derived nucleic acid molecules (CpG DNA). This inhibition also suppressed the expression of neurogenic mediators such as CGRP and Substance P, along with the release of inflammatory factors, thereby alleviating IVD inflammation.^165^

Scaffolds can also be used to regulate the inflammatory microenvironment. Bai and colleagues constructed a ROS-scavenging scaffold in situ, loaded with rapamycin. This scaffold inhibited the mechanistic target of rapamycin complex 1 (mTORC1). Rapamycin alleviated the negative feedback on the PI3K-AKT pathway, enhancing the activation of AKT473 and AKT308, and ultimately promoting M2 macrophage polarization.^238^ These findings underscore the potential of combining drug delivery systems with molecular inhibitors to target specific cellular pathways and achieve therapeutic effects in the treatment of IVD degeneration.

To better match the mechanical properties of the NP, Li and colleagues developed an injectable polysaccharide composite hydrogel with mechanical sensitivity by physically mixing anti-inflammatory fucoidan with DexMA hydrogel. They adjusted the mechanical strength and structure of the DexMA hydrogel to regulate NPC proliferation and ECM protein expression via the CAV1-YAP signaling pathway. Additionally, the incorporated fucoidan helped regulate macrophage polarization towards the M2 phenotype, thereby alleviating inflammation.^239^

However, hydrogels loaded with drugs do not provide effective drug release control. To address this, Cheng and colleagues developed a bioactive hydrogel scaffold made of polyethylene glycol fumarate/sodium methacrylate (OPF/SMA). They embedded two types of microspheres, IL-4-poly (lactic-co-glycolic acid) (PLGA) and KGN-PLGA, within the OPF/SMA hydrogel scaffold to facilitate sequential drug release. IL-4 promotes the transformation of M0/M1 macrophages into M2 macrophages, reducing the secretion of pro-inflammatory factors. Meanwhile, Kartogenin (KGN), a stable non-protein molecule, enhances the homing ability of endogenous host mesenchymal stem cells (MSCs) and also mitigates pain and inflammation by inducing IL-10. Results suggest that the injection of the OPF/SMA + IL-4-KGN-PLGA hydrogel scaffold can protect against disc degeneration and promote tissue regeneration in the damaged segment through early modulation of the inflammatory microenvironment and sustained anti-inflammatory repair of IVDD.^240^

Tissue engineering strategies combine multiple therapeutic approaches, allowing the advantages of different methods to complement each other. In the treatment of IVDD, these strategies can achieve long-term microenvironmental intervention and regulation of cellular activity simultaneously, making them the most clinically translatable therapeutic strategies. As a multidisciplinary integrated approach, tissue engineering provides local support to promote cell growth and tissue regeneration. It can combine drug release with mechanical strength modulation to enhance efficacy by regulating the overall microenvironment, offering various biomaterial options and the potential for personalized treatment.

However, tissue engineering requires a complex design and manufacturing process, and the compatibility and degradability of biomaterials may affect long-term therapeutic outcomes. Despite these challenges, tissue engineering remains the most promising strategy for clinical translation in IVDD treatment.

## Conclusions and perspectives

Recent clinical treatment strategies have not seen significant breakthroughs and remain primarily focused on symptomatic relief. The complex degenerative microenvironment and the inefficient self-regeneration and repair capacity of the IVD make the effective treatment of IVDD a major challenge. Disc herniation and LBP continue to trouble many patients worldwide, causing disability and socio-economic burdens. Currently, clinical treatments for lumbar disc herniation and LBP primarily include surgical and pharmacological approaches, with oral NSAIDs being the mainstay of pharmacological treatment, as recommended as a limited anti-inflammatory treatment option in guidelines issued by the North American Spine Society and the Chinese Orthopedic Association of Spinal Surgery Group.^241–243^

In recent years, the important role of immune cells in various degenerative diseases has been widely recognized. The behavior of macrophages has been thoroughly analyzed, leading researchers to focus their studies on these immune cells. In this review, we analyzed and summarized the role of macrophages in IVDD, finding that macrophages play a crucial role throughout the IVDD process. Influenced by chemokines, macrophages infiltrate the disc tissue via neovascularization, subsequently amplifying the inflammatory response in the IVD through the secretion of inflammatory factors. This process is accompanied by a decline in IVD cell activity and synthesis function, ultimately resulting in disc herniation and LBP. On the other hand, macrophages are involved in the inflammation and post-injury repair of the endplate, causing endplate degeneration, which is also a significant factor in exacerbating IVDD and inducing endplate-derived LBP.

Some studies have also reported the infiltration of other immune cells, such as T cells, B lymphocytes, neutrophils, and natural killer (NK) cells in IVD.^244–248^ Neutrophils and NK cells contribute to IVDD, but research on their roles remains limited.^247,248^ Elucidating the roles of these immune cells could enhance our understanding of the immune microenvironment in IVDD.

Currently, anti-inflammatory treatments targeting macrophages often focus on verifying macrophage polarization within the treatment framework. Understanding the process of macrophage regulation in IVDD, we find that blocking macrophage infiltration caused by chemokines and neovascularization can preemptively prevent inflammation amplification. Leveraging the role of macrophages and their inflammatory factors in regulating ECM remodeling, we might provide new non-surgical treatments for disc herniation. The role of macrophages in endplate inflammation and ossification also makes them potential targets for treating endplate degeneration. By intervening in macrophages and neurogenesis, new therapeutic strategies for LBP may be developed.

Unfortunately, these findings have not yet been effectively translated into clinical practice. First, the safety of treatment strategies in clinical practice requires long-term, systematic evaluation, and only rigorously screened drugs are likely to be recommended for clinical use by guidelines. Additionally, many foundational studies lack long-term assessments, leaving their efficacy uncertain. The rarity of large animal models, such as sheep, means that results may not correspond to human IVDD. Furthermore, current clinical practice favors oral administration, but oral drugs often struggle to achieve effective concentrations in the IVD. Therefore, foundational research frequently relies on intradiscal injection of drugs, and future studies will need to adapt to clinical treatment scenarios.

In other disease models, advanced strategies targeting macrophages are being explored, providing valuable references for precise regulation of these cells. The CRISPR-Cas9 technology allows for the editing of specific genes in target cells, promising to fine-tune of macrophage subtypes. Zhao et al. developed a CRISPR-Cas9 system using Escherichia coli protoplast-derived nanovesicles (NVs) to target Cas9-sgRNA ribonucleoproteins against Pik3cg in tumor-associated macrophages (TAMs), a key molecular switch for polarization, ultimately inducing M2-like TAMs to shift to an anti-tumor M1-like phenotype.^249^ Additionally, targeting macrophage surface receptors enables precise drug delivery. For instance, Li et al. constructed a nanomicelle-hydrogel microsphere that targets the folate receptor (FA receptor) on macrophages. This microsphere releases dexamethasone upon macrophage uptake, promoting M1 macrophage apoptosis and inhibiting polarization, thereby reducing the local “cytokine storm”.^250^ Blocking exosome communication is also a strategy for therapeutic macrophage modulation. Clathrin heavy chain (Cltc) is a gene closely related to endocytosis in macrophages. To reduce macrophage uptake of therapeutic exosomes, Wan et al. created an exosome blocker by encapsulating siRNA against Cltc in exosomes using electroporation. When this blocker was first applied, it significantly reduced the endocytosis capacity of macrophages following uptake. Subsequent application of therapeutic exosomes showed a marked reduction in therapeutic exosome loss, suggesting the possibility of achieving therapeutic effects by modulating exosome communication between macrophages and disc cells.^251^ Additionally, it has been reported that senescent NPCs can release exosomes that induce M1 polarization, suggesting that blocking this process could alleviate inflammation and slow degeneration.^22^ Some macrophage-related pathway inhibitors are widely used in tumor treatment,^252^ their effectiveness in IVDD remains unknown, and these inhibitors may affect other cells, making precise macrophage targeting challenging.

In summary, as our understanding of macrophages deepens, more therapies targeting these cells for IVDD treatment are likely to be developed, which is eagerly anticipated. Continued research into the role of macrophages in IVDD may lead to the discovery of additional clinically translatable therapeutic targets, providing further reference for macrophage-targeted studies.
